# Goethe and Schiller

**Published:** 1880-12

**Authors:** 


### GOETHE AND SCHILLER.

### THE PERSONAL APPEARANCE OF THE TWO GERMAN POETS.

Goethe had dark brown hair and eyes, the latter large and almost preternaturally luminous. His complexion also was more olive than fair, the nose nearly Roman, but with a Greek breadth at the base, and sensitive, dilating nostrils; the mouth and chin, on the sculptor's line, ample, but so entirely beautiful that they seemed smaller than their actual proportions. His face was always more or less tanned; he rarely lost the brand of the sun. In his later years it became ruddy, and a slight increase of fulness effaced many of the wrinkles of age. Stieler's portrait—now in the Goethe mansion—painted when the poet was eighty, expresses an astonishing vital power. Preller once said to me: "There never was such life in so old a man! If a cannon ball had suddenly grazed my head I could not have been more startled than when I heard of his death. I felt sure that he would live to be 150 years old!" If Goethe illustrates as scarcely any other poet (yet we imagine both Homer and Shakespeare to have possessed the same) the perfect accord of intellectual and physical forces, Schiller is equally remarkable as an example of a mind triumphing over incessant bodily weaknesses and torments. During fourteen years he never knew a day of complete unshaken health. He was fair and freckled, with so delicate a skin that the slightest excitement of his blood blushed through it. His thin, aggravated, aquiline nose was so conspicuous that he often laughingly referred to it as the triumphant result of constant pinching and pulling during his school-days. His chin was almost equally prominent, giving him what his sister Christophine called "a defiant and spiteful under-lip." His shock of hair, not parting into half curls like Goethe's, but straight and long, was of a yellow-brown hue, "shimmering into red," as Caroline von Wolzogan poetically says. The picture of him touches our sympathies, as his bust or statue always does—perhaps because he represents suffering and struggle so palpably. Beside him Goethe seems to stand crowned by effortless achievement. But what a pair they are! Rietschel's great success in his statues lies in his subtle expression of their noble friendship. Goethe's hand on Schiller's shoulder, and the laurel wreath which the hands of both touch, in such wise that you cannot be sure which gives or which takes, symbolize a reality far too rare in the annals of literature.

